# Differential expression analysis of mRNAs, lncRNAs, and miRNAs expression profiles and construction of ceRNA networks in PEDV infection

**DOI:** 10.1186/s12864-022-08805-0

**Published:** 2022-08-13

**Authors:** Xiaojie Shi, Qi Zhang, Jingjing Wang, Yuting Zhang, Yuchao Yan, Yi Liu, Naling Yang, Quanqiong Wang, Xingang Xu

**Affiliations:** College of Veterinary Medicine, Northwest A&F University, Yangling, Xianyang, 712100 Shaanxi China

**Keywords:** PEDV, lncRNA, Functional enrichment, Signaling pathway, ceRNA network

## Abstract

**Background:**

Porcine Epidemic Diarrhea Virus (PEDV) is a coronavirus that seriously affects the swine industry. MicroRNAs and long noncoding RNAs are two relevant non-coding RNAs (ncRNAs) class and play crucial roles in a variety of physiological processes. Increased evidence indicates a complex interaction between mRNA and ncRNA. However, our understanding of the function of ncRNA involved in host-PEDV interaction is limited.

**Results:**

A total of 1,197 mRNA transcripts, 539 lncRNA transcripts, and 208 miRNA transcripts were differentially regulated at 24 h and 48 h post-infection. Gene ontology (GO) and KEGG pathway enrichment analysis showed that DE mRNAs and DE lncRNAs were mainly involved in biosynthesis, innate immunity, and lipid metabolism. Moreover, we constructed a miRNA-mRNA-pathway network using bioinformatics, including 12 DE mRNAs, 120 DE miRNAs, and 11 pathways. Finally, the target genes of DE miRNAs were screened by bioinformatics, and we constructed immune-related lncRNA-miRNA-mRNA ceRNA networks. Then, the selected DE genes were validated by qRT-PCR, which were consistent with the results from RNA-Seq data.

**Conclusions:**

This study provides the comprehensive analysis of the expression profiles of mRNAs, lncRNAs, and miRNAs during PEDV infection. We characterize the ceRNA networks which can provide new insights into the pathogenesis of PEDV.

**Supplementary Information:**

The online version contains supplementary material available at 10.1186/s12864-022-08805-0.

## Background

Porcine epidemic diarrhea (PED) is one of the infectious intestinal diseases of swine, manifested by severe diarrhea, vomiting, and dehydration [1]. Porcine epidemic diarrhea virus (PEDV), the etiological agent of PED, was originally identified in the United Kingdom in the 1970s [2]. PEDV is a single-stranded positive-stranded RNA virus that is a member of the *Coronavirus* family. The genome of PEDV is roughly 28 kb and contains seven open reading frames (ORF), including the ORF1a, ORF1b, S, ORF3, E, M, and N genes [3]. In 2010, the outbreak of PED caused by highly virulent PEDV strains spread in China, resulting in a mortality rate of nearly 100% in suckling piglets and huge economic losses [4–6]. Due to the high frequency of mutations in the PEDV genome, existing vaccines are ineffective in protecting pigs infected with highly dangerous strains [7]. Although the pathogenesis of PEDV has been studied, the mechanisms of host-PEDV interactions are still limited.

Non-coding RNAs (ncRNAs), such as small ncRNAs (sncRNAs) and long ncRNAs (lncRNAs), are transcribed from a large percentage of host genomes [8]. More and more studies have indicated that ncRNAs play crucial roles in many biological processes, including immune responses, post-transcriptional regulation, apoptosis, and autophagy [9–11]. MicroRNAs (miRNAs) are a type of abundant sncRNAs (~ 22nt long) that negatively regulate gene expression by binding to the 3’UTR of the mRNA [12]. It is well known that miRNAs are closely related to the infection and proliferation of various viruses [13, 14]. For example, miR-30c-5p inhibits SOCS1 expression by targeting the SOCS1 3'UTR region to facilitate PEDV replication [15]. MiR-129a-3p activates the NF-κB pathway by targeting the 3'UTR of EDA, thereby inhibiting the replication of PEDV [16].

LncRNAs are a class of RNAs that are more than 200 nucleotides (nt) in length and have no protein-coding capability [17]. Compared with mRNA, lncRNA is more tissue-specific and less conservative [18]. Several studies have shown that viral infection induces lncRNAs to regulate antiviral innate immunity [19]. LncRNA NEAT1 exerts an antiviral effect by promoting IFN response during Hantavirus infection [20]. On the other hand, another study has shown that lncRNA-CMPK2 acts as a negative regulator of IFN response to promote HCV replication [21]. In recent studies, the competing endogenous RNA (ceRNA) theory has been reported as a new regulatory mechanism between lncRNA and mRNA [22]. LncRNAs act as miRNA sponges to indirectly regulate the degradation or translational inhibition of target mRNA [22]. So far, ceRNA theory has become the mainstream method for studying lncRNA-miRNA-mRNA interaction and has been verified by many physiological processes. Lnc-ISG20 binds to miR-326 to enhance the expression of ISG20 and inhibit IAV replication [23]. However, there are no studies on the lncRNA-miRNA-mRNA network during PEDV infection.

Recent studies have analyzed the changes of ncRNA expression profiles during PEDV infection [1, 24, 25]. However, the regulatory mechanism of ceRNA in PEDV-infected cells remains unclear. In this study, we performed the systematic analysis of mRNA, lncRNA, and miRNA expression profiles at two different time points during PEDV infection. We analyzed differentially expressed genes, identified the target genes of DE lncRNAs and DE miRNAs, and characterized the mRNA-miRNA-pathway network and immune-related ceRNA network. Our study aims to explore the potential role of ncRNAs during PEDV infection and expand the understanding of the host-virus interactions.

## Results

### Overview of RNA-sequencing

**Fig. 1:**
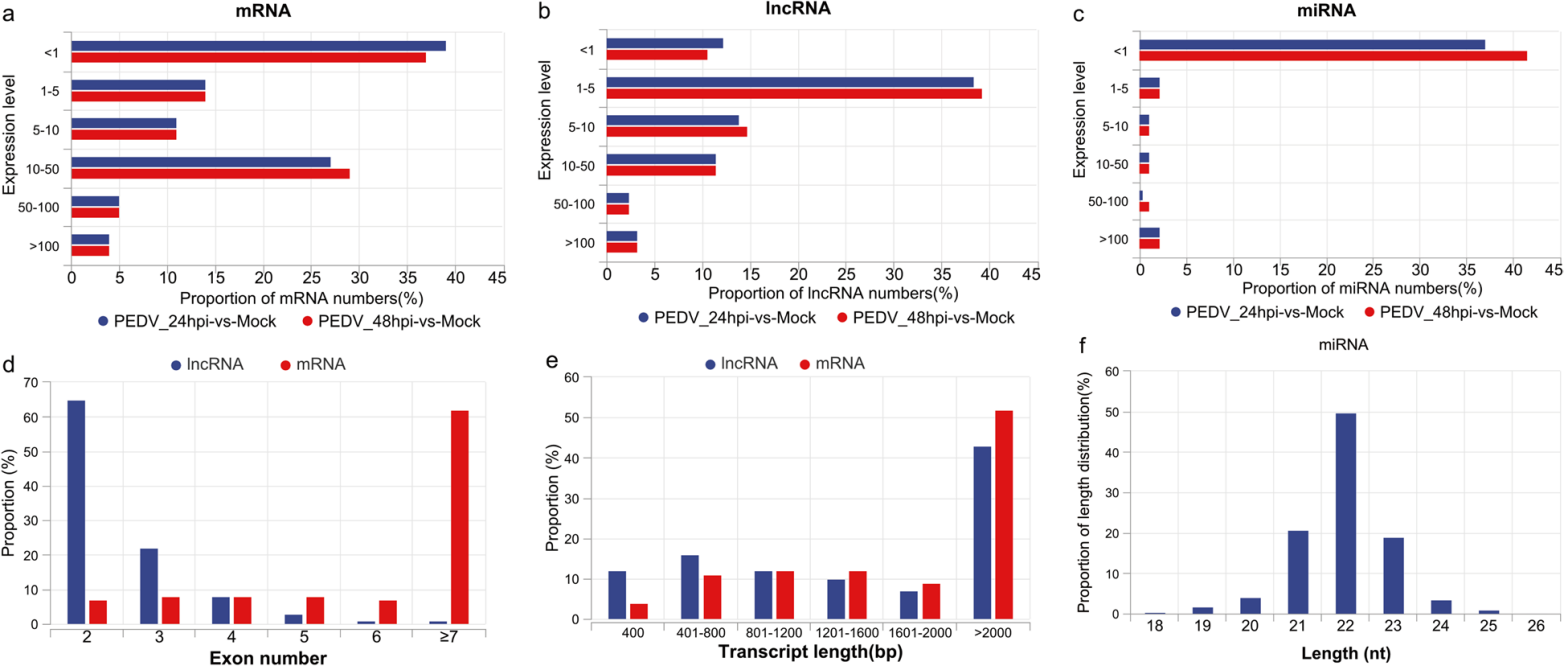
Overview of RNA sequencing. The distribution of mRNA (**a**), lncRNA (**b**), and miRNA (**c**) expressions at different times following viral infection were represented by histograms. (**d**) Exon number distribution of mRNAs and lncRNAs. Length distribution of mRNAs, lncRNAs (**e**), and miRNAs (**f**)

To identify the ncRNAs (lncRNA and miRNA) and protein-coding transcripts associated with viral infections, 18 RNA-seq libraries were sequenced. A total of 995 M raw reads were obtained and an average of 983.63 M clean reads per library was produced (Table S1). In addition, at least 89% of transcripts in each library were covered by reads (Table S2). Meanwhile, approximately 71.33 M clean reads were obtained from 9 small RNA sequencing libraries (Table S3). In this study, we identified 17,500 mRNAs, 5,748 lncRNAs, and 659 miRNAs. The RNA analysis suggested that, except for miRNA, the distribution of mRNA and lncRNA in each treatment group was essentially the same (Fig. 1a-c, Fig. S1). The proportion of miRNA expression < 1 was greater than 41% in the 48hpi-vs-Mock group. Following that, the properties of mRNA and lncRNA transcripts were assessed. The number of mRNA exons was noticeably greater than that of lncRNA exons. The findings revealed that mRNAs with more than 7 exons exceeded 60%, while lncRNAs mostly contained only two or three exons (Fig. 1d). The average length of these mRNAs was longer than the average length of lncRNAs, and the majority of the lncRNAs were longer than 2000 bp (Fig. 1e). Furthermore, miRNA length analysis revealed that it was commonly distributed at 22nt (Fig. 1f).

### Identification and analysis of differentially expressed mRNAs

**Fig. 2:**
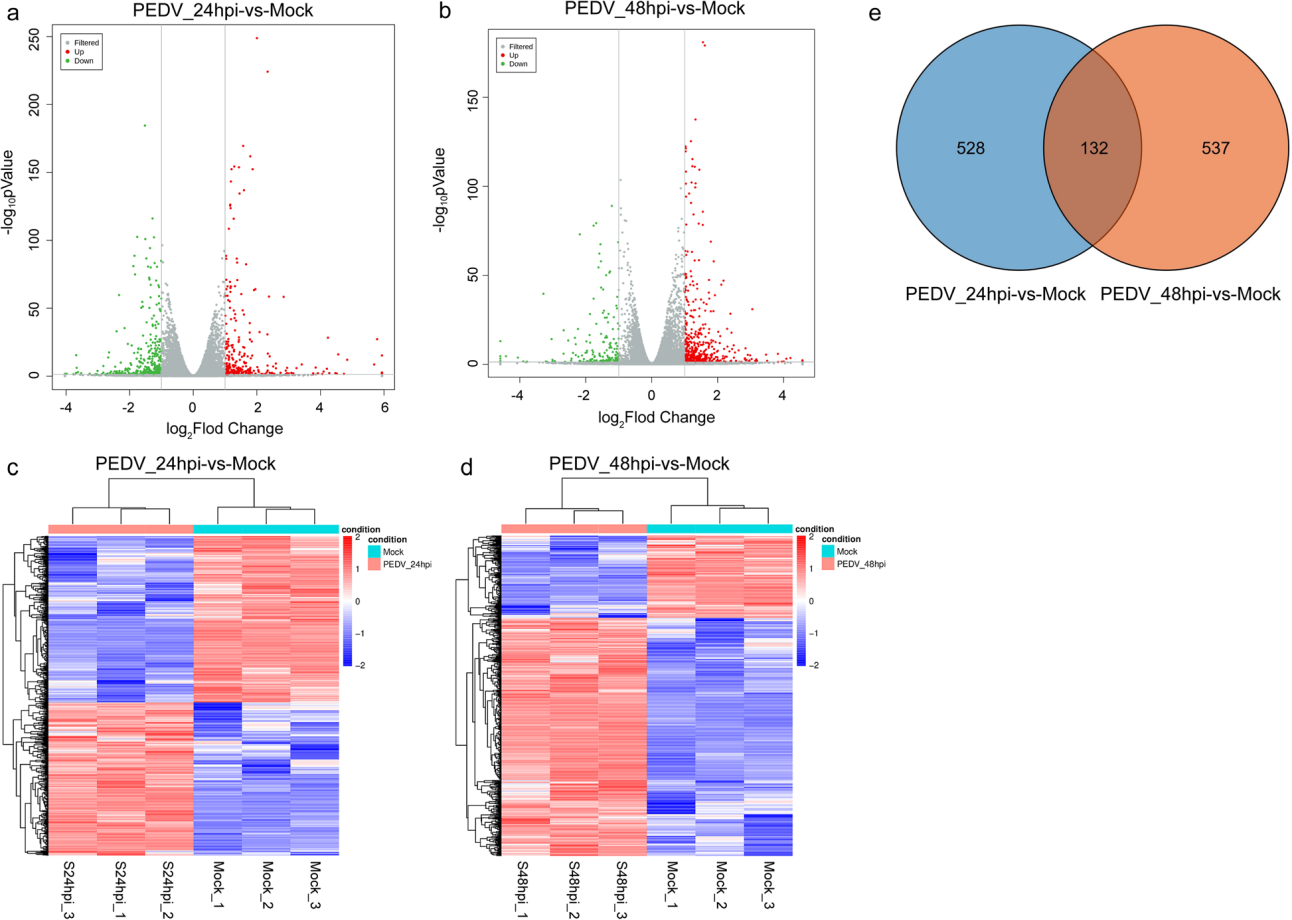
Identification and characterization of mRNAs. (**a**, **b**) Volcano plots of DE mRNAs between the mock-infected and PEDV-infected groups. The red and green dots represent up-regulated and down-regulated genes, respectively. (**c**, **d**) Heat map showing DE mRNAs expression levels. Individual samples are represented by columns, while genes with significant expression differences between the two groups are represented by rows. (**e**) Venn diagram for the intersection of DE mRNAs between the two groups

**Fig. 3:**
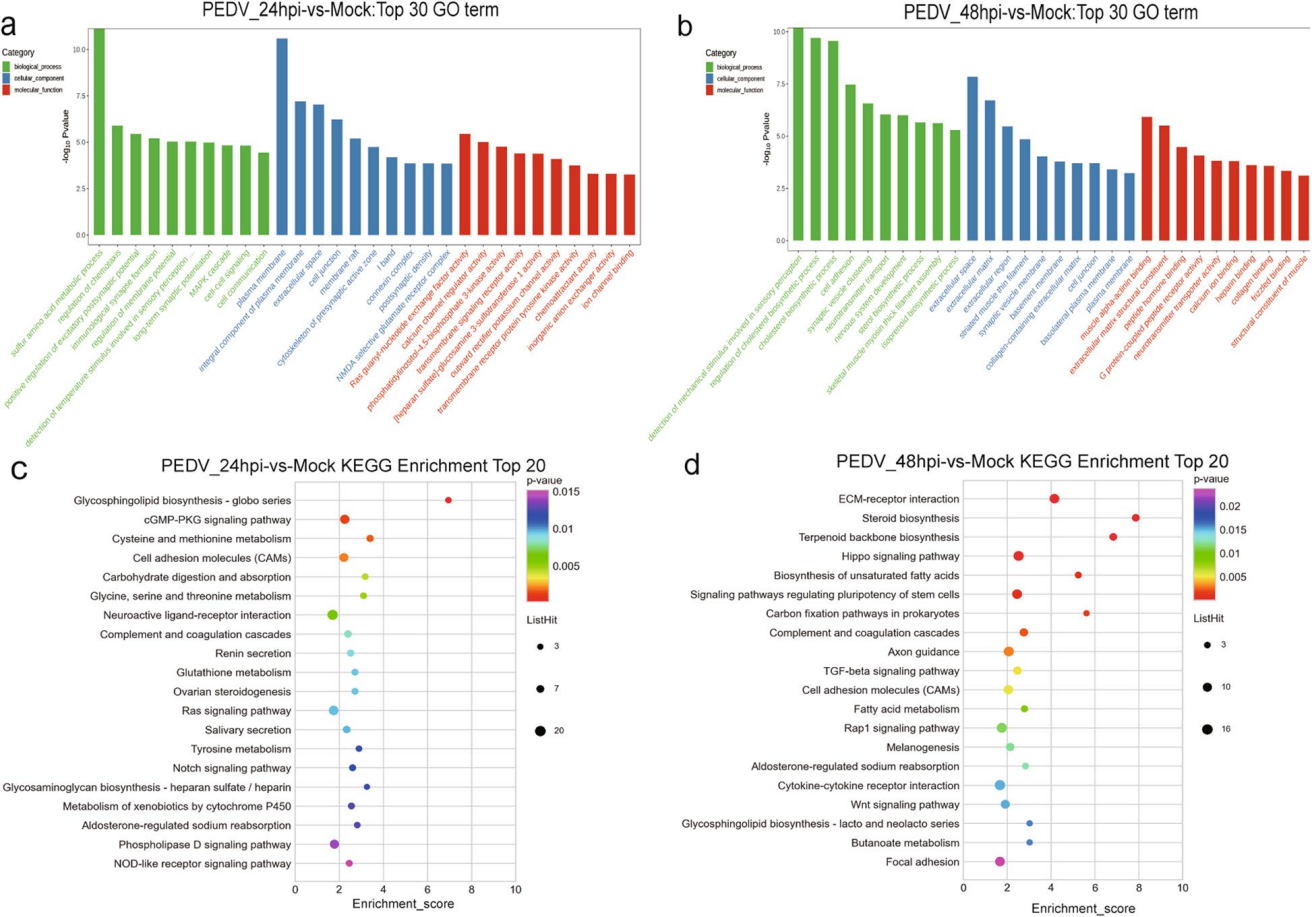
Functional enrichment analysis of DE mRNAs. (**a**, **b**) The top 30 significantly enriched GO terms. (**c**, **d**) The top 20 significantly enriched KEGG pathways

In this study, we identified 660 and 669 DE mRNAs in two groups, including 813 up-regulated (61.17%) and 516 down-regulated (38.83%) (Fig. 2a and b, Table S4). Next, the hierarchical cluster analysis revealed that the expression profiles of mRNAs changed significantly during PEDV infection, and 132 common DE mRNAs were found between the two groups through Venny 2.1 (Fig. 2c-e). GO enrichment analysis suggested that these DE mRNAs were significantly enriched in the biosynthetic process, metabolic process, cell–cell signaling, and binding (Fig. 3a and b). Meanwhile, KEGG analysis indicated that several DE mRNAs were enriched in signal transduction pathways, including the Rap1 signal, Wnt signal, cGMP-PKG signal, TGF-beta signal, and NOD-like receptor signaling pathway (Fig. 3c and d).

### Identification and analysis of miRNAs

**Fig. 4:**
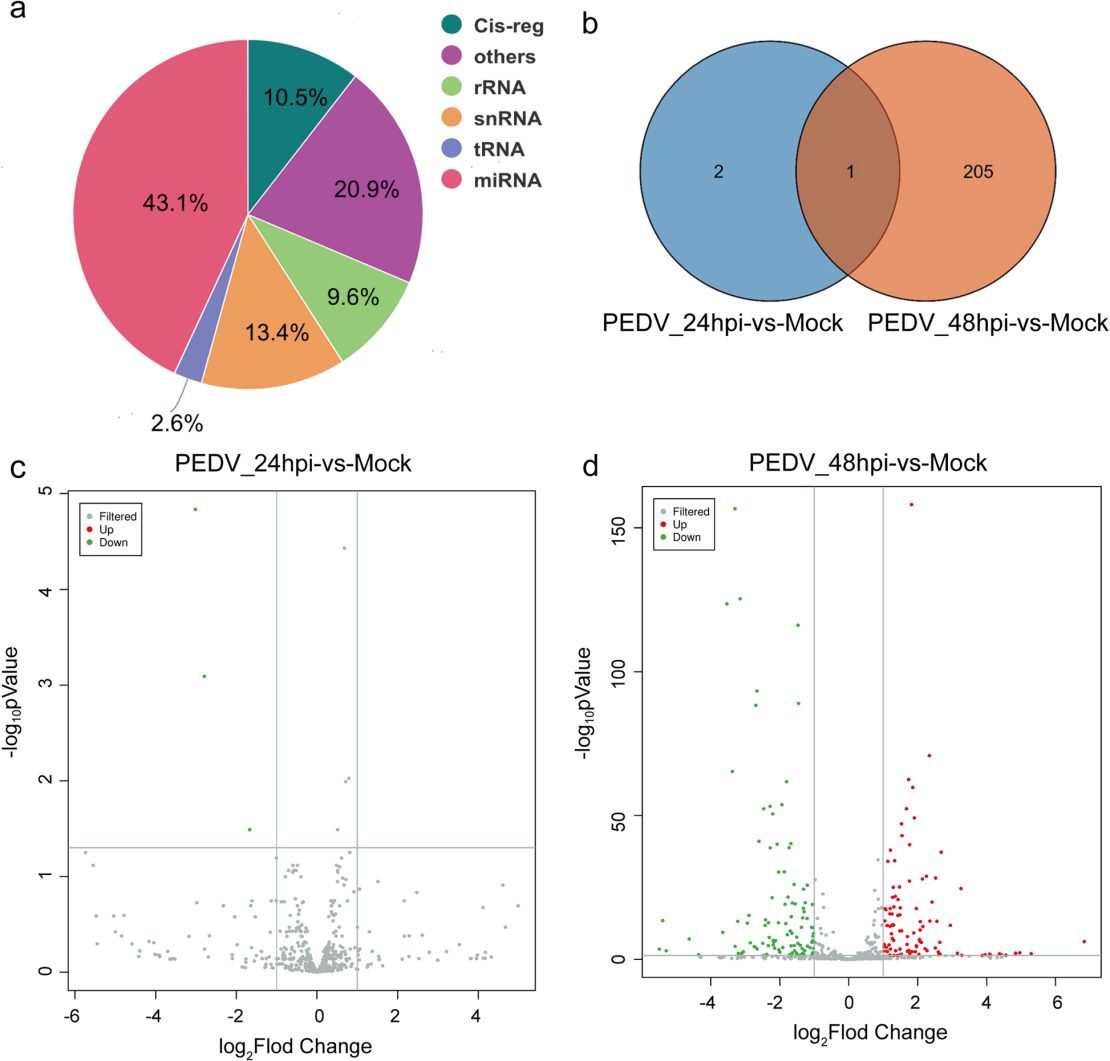
Identification and characterization of miRNAs. (**a**) Portions of small RNA types in the clean reads. (**b**) Venn diagram for the intersection of DE miRNAs between the two groups. (**c**, **d**) Volcano plots of DE miRNAs between the mock-infected and PEDV-infected groups. Up-regulated and down-regulated genes are represented by red and green dots, respectively

To analyze the characteristics of miRNAs during PEDV infection, miRNA expression levels were compared in different groups. We detected 43.1% mature miRNAs after comparing the clean reads to the GenBank, Rfam database, and reference genome. (Fig. 4a). A total of 616 and 641 miRNAs were detected from 24hpi-vs-Mock and 48hpi-vs-Mock, respectively. Among them, 598 miRNAs were co-expressed, while 18 and 43 miRNAs were specifically expressed in the two groups. According to fold change > 2 and *P*-value < 0.05, there were 3 and 206 DE miRNAs, including 100 up-regulated (31.9%) and 109 down-regulated (68.1%) were identified (Fig. 4b-d, Fig. S2a and b, Table S5). A total of 7,352 target mRNAs for DE miRNAs were identified, and we identify the potential physiological functions of DE miRNAs through GO and KEGG enrichment analyses (Fig. S3a-d).

### Construction of miRNA-mRNA-pathway network

**Fig. 5:**
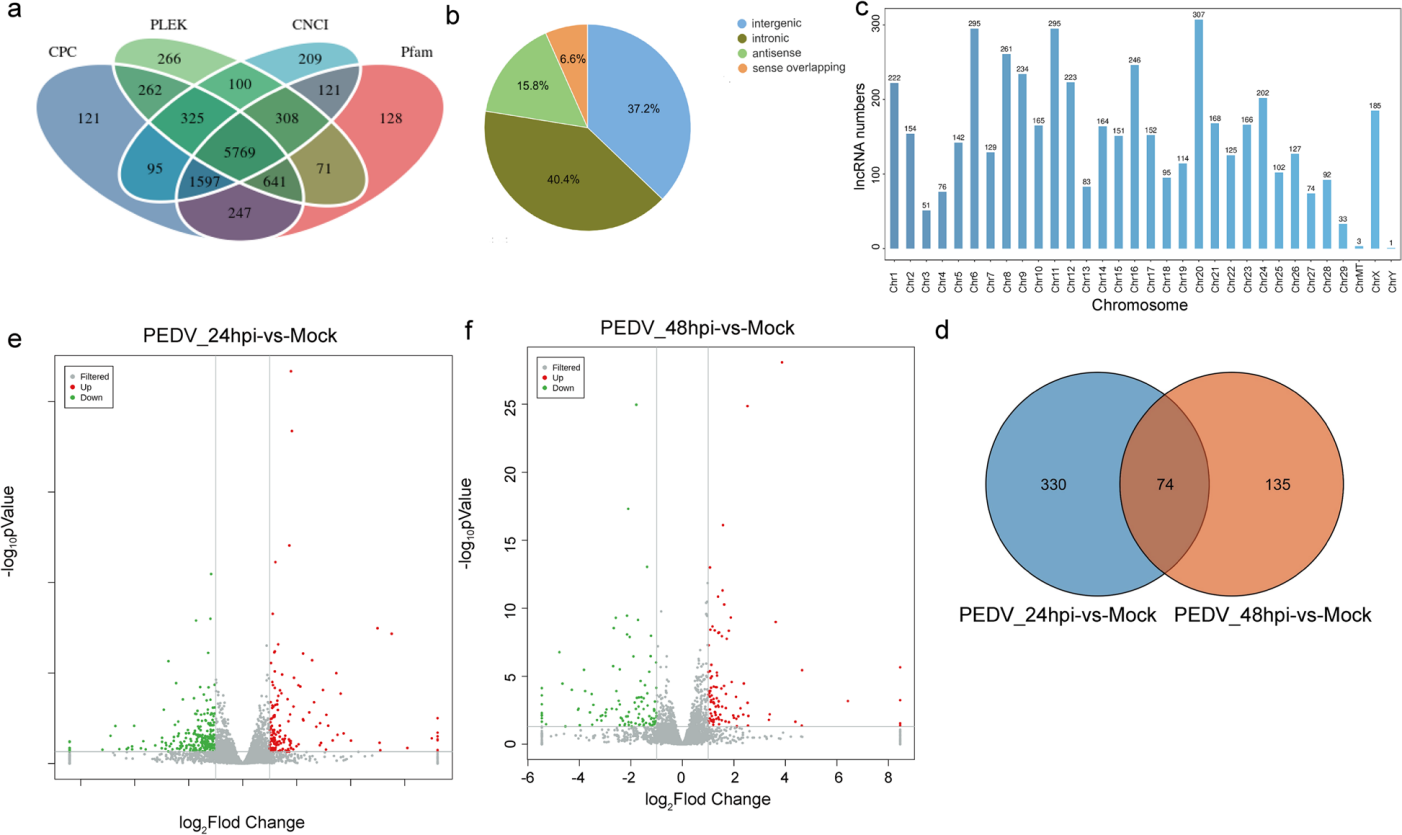
The miRNA-mRNA-pathway network. The ellipse, round rectangle, and diamond symbols represent mRNA, miRNA, and the pathway, respectively. Up-regulated and down-regulated genes are represented by red and green, respectively

Among intersecting genes, 12 genes related to cellular immunity and cell growth along with their corresponding 120 DE miRNAs were selected (Table 1). CSF2 (Colony Stimulating Factor 2), BMP2 (Bone Morphogenetic Protein 2), WNT11 (Wnt Family Member 11), SFN (Stratifin), and TNFAIP3 (TNF Alpha Induced Protein 3) genes are involved in a variety of pathways. To visualize the interactions and further explore the function of the DE mRNAs and their corresponding DE miRNAs, the miRNA-mRNA-pathway network was constructed using the data in Table 1 (Fig. 5).

**Table 1:** Twelve intersection genes and their corresponding pathways and differentially expressed miRNAs

DE mRNAs	Pathway	Numbers of differentially expressed miRNAs
IL2RB	Jak-STAT signaling pathway	4
CSF2	Jak-STAT signaling pathway, TNF signaling pathway	0
ID3	TGF-beta signaling pathway	11
BMP2	TGF-beta signaling pathway, MAPK signaling pathway	27
FGF21	MAPK signaling pathway	3
DUSP5	MAPK signaling pathway	17
MEFV	NOD-like receptor signaling pathway	7
TNFAIP3	NF-kappa B signaling pathway, NOD-like receptor signaling pathway, TNF signaling pathway	28
WNT11	mTOR signaling pathway, Wnt signaling pathway	9
SFN	p53 signaling pathway, Cell cycle	1
PRKN	Ubiquitin mediated proteolysis	9
TRBV	NF-kappa B signaling pathway	4

### Identification and analysis of lncRNAs

**Fig. 6:**
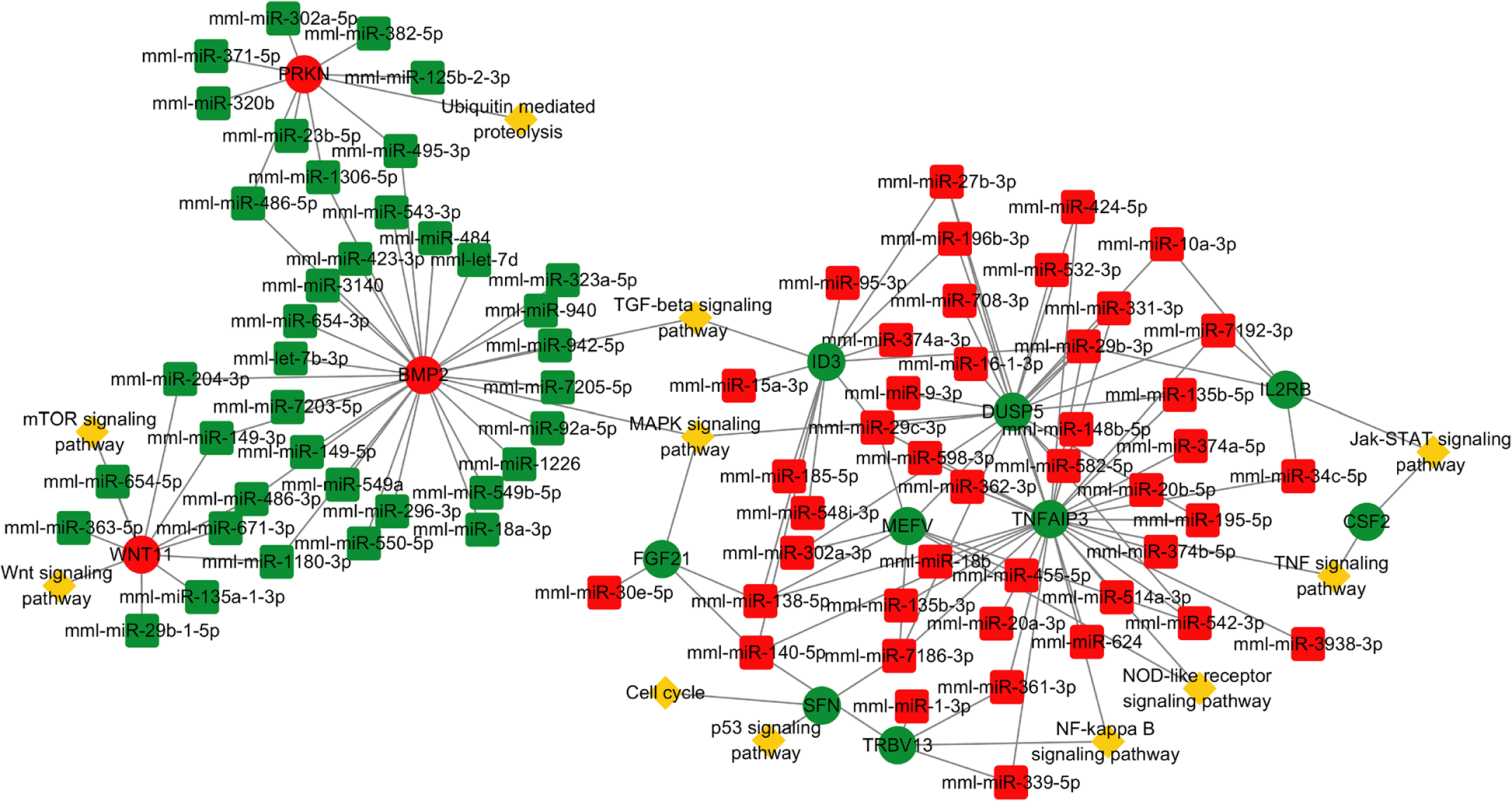
Identification and characterization of lncRNAs. (**a**) Screening of non-coding lncRNAs by using CPC, Pfam, PLEK, and CNCI. (**b**) Distribution of the four types of lncRNAs. (**c**) Chromosome distribution of lncRNAs. (**d**) Venn diagram for the intersection of DE lncRNAs between the two groups. (**e**, **f**) Volcano plots of DE lncRNAs between the mock-infected and PEDV-infected groups. Up-regulated and down-regulated genes are represented by red and green dots, respectively

In this study, CNCI, CPC, Pfam-scan, and PLEK were used to remove potential coding transcripts, and 5,769 lncRNAs were obtained. Through further filtering, we finally identified 5,748 lncRNAs, which included 37.2% intergenic lncRNAs and 40.4% intronic lncRNAs (Fig. 6a and b). Most of the lncRNA transcripts were found on chromosome 20, however, few lncRNAs were found on chromosome Y (Fig. 6c). A total of 404 and 209 DE lncRNAs were detected in 24hpi-vs-Mock and 48hpi-vs-Mock, respectively, including 269 up-regulated (31.9%) and 344 down-regulated (68.1%) (Fig. 6d-f, Fig. S2c and d, Table S6). In addition, 1,197 protein-coding genes were found to exhibit significant correlations with DE lncRNAs in expression, of which 1,169 were detected as trans-regulated target genes. As shown in Fig. S4, GO terms analysis and KEGG analysis was performed.

### Construction of ceRNA network

**Fig. 7:**
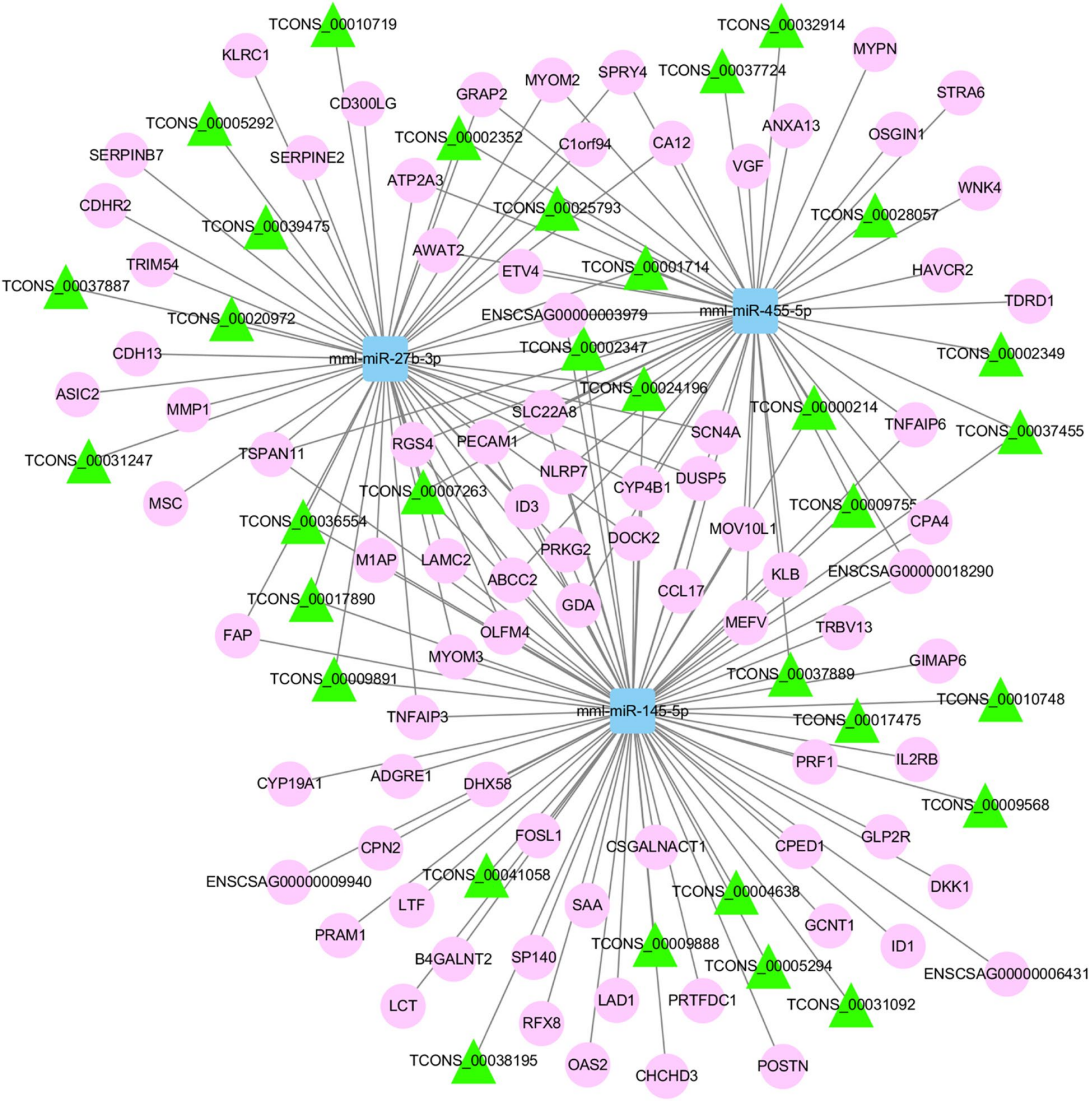
An overview of the lncRNA-miRNA-mRNA ceRNA network. The ellipse, triangle, and round rectangle symbols represent mRNA, lncRNA, and miRNA respectively

We used miRanda (v3.3a) to identify the targets of each DE miRNA from DE mRNA and DE lncRNA transcripts. We selected 3 DE miRNAs important in the innate immunity and built a ceRNA network by incorporating miRNA-target interactions. The ceRNA network consisted of 3 DE miRNAs, 82 DE mRNAs, 32 DE lncRNAs, and 171 edges (Fig. 7). The DE mRNAs in the ceRNA network included TNFAIP3, MEFV (MEFV innate immunity regulator, pyrin), TRBV13 (T Cell Receptor Beta Variable 13), DUSP5 (Dual Specificity Phosphatase 5), ID3 (Inhibitor of DNA Binding 3, HLH Protein), TRIM54 (Tripartite Motif Containing 54), and IL2RB (Interleukin 2 Receptor Subunit Beta), etc. Functional enrichment analysis revealed that these DE mRNAs were involved in several immune-related pathways, including apoptosis, NOD-like receptor signaling, PI3K-Akt, NF-kappa B signaling, and RIG-I-like receptor signaling pathway. In addition, we also constructed an extensive ceRNA network to reveal the relationships among all DE genes (Fig. S5).

### qRT-PCR validation of gene expression

**Fig. 8:**
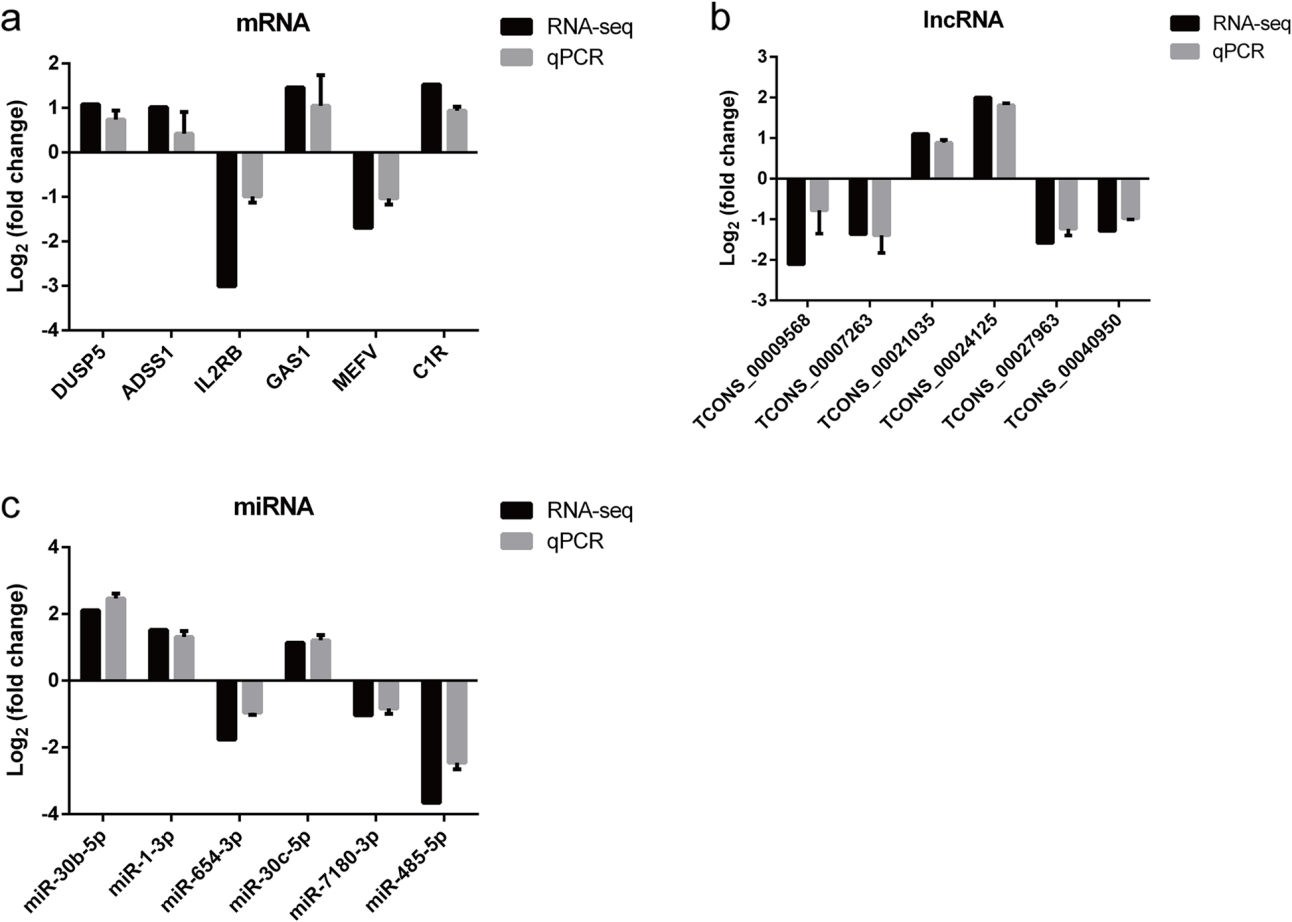
Validation of RNA-seq results by qRT-PCR analysis. (**a**) The expression levels of DE mRNAs were validated by qRT-PCR. (**b**) The expression levels of DE lncRNAs were validated by qRT-PCR. **(c**) The expression levels of DE miRNAs were validated by qRT-PCR. Log_2_ fold change was expressed as mean ± SD

To further verify the RNA-seq data, we randomly identified 6 DE mRNAs (ADSS1, GAS1, DUSP5, MEFV, IL2RB, and C1R), 6 DE lncRNAs (TCONS_00009568, TCONS_00007263, TCONS_00021035, TCONS_00024125, TCONS_00027963, and TCONS_00040950), and 6 DE miRNAs (mml-miR-30b-5p, mml-miR-1-3p, mml-miR-30c-5p, mml-miR-654-3p, mml-miR-7180-3p, and mml-miR-485-5p) by qRT-PCR. The results showed the expression pattern of these genes was consistent with those obtained by the RNA-Seq (Fig. 8).

## Discussion

PEDV caused huge economic losses to the livestock industry, and commercial vaccines cannot provide complete protection for pigs [7]. Although research on PEDV has made great progress in recent decades, the pathogenic mechanism of PEDV has not been fully studied. Recent research has demonstrated that lncRNAs can act as miRNA sponges, regulating the expression of host genes and hence influencing virus replication. However, the characterization of the ceRNA mechanism in host cells during PEDV infection has not been elucidated. In this study, we detected changes in the expression profiles of mRNAs, lncRNAs, and miRNAs in PEDV-infected MARC-145 cells. The highest number of DE lncRNAs was discovered at 48 hpi, and there were more up-regulated genes were identified. These characteristics reveal that the host response to PEDV infection involves activating a large number of genes. Comparative analysis of the genomic characteristics of mRNAs and lncRNAs reveals that lncRNAs have shorter transcripts, fewer exons, and lower expression levels, which is consistent with the results of transcriptome analysis in different species [26–28]. In addition, the expression changes of randomly selected differentially expressed genes were confirmed by qRT-PCR analysis, indicating the accuracy of the sequencing results.

Recent studies suggest a close relationship between cholesterol metabolism and innate immunity [29–31]. In addition, the ATP binding cassette (ABC) transporters play a key role in many metabolic processes and contribute to resisting the penetration of xenobiotic [32]. By analyzing these DE mRNAs, we found that the expression levels of ABCG1, ABCC3, LPIN1, DHCR7, HMGCR, and INSIG1 genes were significantly up-regulated during PEDV infection. These genes related to cholesterol metabolism were significantly up-regulated, suggesting that the host may resist PEDV infection by regulating lipid metabolism. We also studied the changes in the expression of genes associated with immunity. The sequencing results revealed that toll-like receptors (TLR2 and TLR10), some inflammatory factors (IL24, IL1RN, and IL37), and several antiviral genes such as IFIT1, ISG15, and TNK1 were differentially expressed. These up-regulated DE mRNAs were mainly focused on the immune-related pathways, such as Toll-like receptor signaling, p38 MAPK signaling, IFN signaling, and TNF signaling pathway. The data presented here may indicate that the host resists viral infection by up-regulating the genes described above in 48 h. As we all know, PEDV can use a variety of strategies to antagonize innate immune responses to ensure self-proliferation. However, no significant changes in mRNA levels of typical immune signaling factors such as MAVS, TRAF3, and IRF3 were detected during the early stages of PEDV infection. Therefore, we hypothesize that this may be one of the ways PEDV evades the host innate immune response. Further in vivo and in vitro studies are required to confirm these findings.

At present, only two studies have focused on lncRNA profile variation in PEDV-infected cells. Yu et al. analyzed PEDV-infected Vero cells at different infection time points and DE lncRNAs were screened [33]. Chen et al. compared the lncRNA profiles in PEDV infected IPEC-J2 cells at different time points and obtained the DE lncRNAs related to immunity [25]. There are several differences between our study and other studies. Firstly, we conducted a synthetic analysis of the mRNA, lncRNA, and miRNA expression profiles of PEDV-infected cells and performed KEGG and GO enrichment analysis. Secondly, we predicted the DE mRNAs and DE lncRNAs interacting with DE miRNAs through the database, and the ceRNA network was built. Subsequently, the enrichment analysis of DE mRNAs and DE lncRNAs showed the enrichment of cell growth, steroid synthesis, fatty acid synthesis, and other lipid metabolism pathways, which is different from previous reports.

In our study, we found that the position of lncRNA in the genome is non-random, which indicates a connection between the lncRNA function and genomic position. Ørom et al. found that a group of human lncRNAs can perform enhancer-like functions and increase the expression of neighboring protein-coding genes through cis-acting [34]. Furthermore, another report clarified that lncRNA-mediated cis-regulation of adjacent gene transcription is common in mammalian gene regulation [35]. Therefore, knowledge of the cis-regulation of lncRNA is essential to reveal its biological effects. In our study, several lncRNAs (TCONS_00031360, TCONS_00030751, TCONS_00005777, TCONS_00021191, etc.) were involved in the cis-regulation of autophagy-related genes during PEDV infection. Our data also indicated that some lncRNAs targeted immunity-related genes such as DDIT3, TXNIP, and FOSL1. Although lncRNA-mediated cis-regulation is ubiquitous, some lncRNAs can also function in trans. For example, the expression of several lncRNAs (TCONS_00039521, TCONS_00019044, TCONS_00024478, and TCONS_00021764) was significantly increased, and their target genes were ADA2, FADS1, MEA1, ADGRE1, MAN1C1, PNPLA3, and RND2, which were located in the trans-position.

Several studies have demonstrated the extensive involvement of miRNAs in viral pathogenesis as well as in multiple immune responses. It is universally known that IRF3 is a member of the IRF family that can induce the expression of type I IFN to regulate the antiviral innate immune response. Interestingly, our sequencing data showed that the expression levels of miR-296-3p, miR-486-3p, miR-940, and miR-1306-5p, whose target gene is IRF3, were significantly down-regulated. Therefore, we hypothesize that the host innate immune system was effectively activated to down-regulate miRNA expression in response to viral infection. Furthermore, several DE miRNAs targeting immune-related genes were identified, but with different expression trends. This finding also indicates that these miRNAs are differentially regulated and perform diverse functions during viral infection. In addition, we found that the target genes of several DE miRNAs were enriched in lipid metabolism and synthesis pathways, which demonstrated that miRNAs may be involved in the regulation of lipid.

More and more research has shown that lncRNAs can act as miRNA sponges, and the construction of ceRNA networks is a new strategy to investigate gene function. Therefore, we selected 3 DE miRNAs to build a ceRNA network to explore the exact roles and mechanisms of DE lncRNAs. Integration analysis of the lncRNA-associated ceRNA network revealed the functions of lncRNAs and their roles in cell proliferation and innate immune response. We predicted many lncRNA-miRNA-mRNA regulatory axes that may be involved in the regulation of PEDV replication, for example lnc_00037724-miR-455-5p-MEFV, lnc_00036554-miR-27b-3p-ID3, and lnc_00010748-miR-145-5p-TNFAIP3. It has been reported that MEFV regulates innate immunity by degrading multiple inflammasome components, including CASP1, NLRP1, and NLRP3 [36]. Other studies have documented that ID3 is involved in several biological processes, including cell proliferation, senescence, and apoptosis [37]. TNFAIP3 is a ubiquitin editing enzyme with immunosuppressive properties, preventing NF-κB activation and TNF-mediated apoptosis [38, 39]. Furthermore, several lncRNA-miRNA-mRNA axes with similar expression patterns were identified, which were enriched in different biological processes. Although the results predicted by the software need to be further investigated, our findings provide new evidence for the involvement of lncRNAs in host antiviral immunity.

## Conclusions

In summary, our study provides the systematic description of the expression profiling of mRNAs, lncRNAs, and miRNAs. A total of 17,500 mRNAs, 5,748 lncRNAs, and 659 miRNAs were identified. In addition, we constructed a ceRNA regulatory network which contained 3 DE miRNAs, 82 DE mRNAs, and 32 DE lncRNAs based on the co-expressed transcripts. Therefore, the data acquired in this study provides a beneficial reference for understanding the interaction between the host and PEDV.

## Methods

### Cells culture and virus infection

MARC-145 cells were maintained in Dulbecco’s modified Eagle’s essential medium (DMEM; Hyclone, USA) supplemented with 10% fetal bovine serum (FBS; Gibco, USA) and cultured at 37 °C with 5% CO_2_. PEDV strain CH/SXYL/2016 (GenBank: MF462814.1) used in this study was isolated as previously described [40]. The cells were infected with PEDV at an MOI of 1 and incubated in DMEM containing 2 μg/mL Trypsin for 1 h at 37 °C with 5% CO_2_. After incubation, the cells were washed with phosphate-buffered saline (PBS) and cultured in DMEM supplemented with 2% FBS.

### RNA extraction

The kinetics of PEDV replication in Marc-145 cells showed that the viral RNA levels peaked at 48 hpi and decreased thereafter [41].Therefore, the samples from 24 and 48 hpi were selected for RNA sequencing. MARC-145 cells were divided into three groups: cells infected with PEDV for 24 h (S24hpi_1, 2, 3), cells infected with PEDV for 48 h (S48hpi_1, 2, 3), and mock-infected cells as controls (Mock_1, 2, 3). Three biological replicates were performed. According to the manufacturer's instructions, the total RNA of PEDV-infected cells and uninfected cells was extracted using the TRIzol® reagent (Invitrogen, USA). RNA quality and concentration were tested by NanoDrop 2000 (Thermo Scientific, USA) and Agilent 2100 Bioanalyzer (Agilent Technologies, USA). The samples with RNA Integrity Numbers (RIN) ≥ 7 were subjected to the subsequent analysis.

### Library construction and sequencing

A total of 1 μg RNA from each sample was used as input for RNA-seq library preparation. According to the manufacturer’s instructions, ribosomal RNA (rRNA) was removed using an Epicenter Ribo-Zero rRNA Removal Kit (Illumina, USA), and small RNA libraries were constructed using TruSeq Small RNA Sample Prep Kit (Illumina, USA). Meanwhile, sequencing libraries of mRNA and lncRNA were generated using NEBNext® Ultra™ II Directional RNA Library Prep Kit for Illumina® (NEB, USA). Thereafter, the library products were assessed for purity using an Agilent 2100 Bioanalyzer and then sequenced using the Illumina HiSeq X Ten platform by OE Biotech Co.

### Analysis of differentially expressed mRNAs

First, low-quality reads were removed from the raw data using Trimomatic (v0.36) [42] to obtain clean reads. Then, the clean reads were mapped to the Chlorocebus sabaeus genome using HISAT2 (v2.2.1) [43], and the mapped reads of each sample were assembled using StringTie (v2.1.1) [44]. FPKM [45] of each gene was calculated using Cufflinks (v2.2.1) [46], and the read counts of each gene were obtained by HTSeq-count (v0.9.1) [47]. Analysis of DE mRNAs was performed using the DESeq R package [48]. Fold change > 2 and *P*-value < 0.05 were set as the thresholds for significantly differential expression.

### miRNA identification and analysis

Cutadapt (v1.14) was used to remove adapter dimers, repetitive sequences, and sequences shorter than 18 nucleotides in the raw data. The clean data was aligned by ACGT101-miR (v4.2) to eliminate RNA families (rRNA, tRNA, snRNA, snoRNA) and repeat sequences. Then, the clean reads were mapped to the miRBase database (v22.0) to identify known miRNAs and novel miRNAs. The miRNA expression was calculated and normalized as transcripts per million (TPM), and DE miRNAs were selected according to fold change > 2 and *P*-value < 0.05.

### lncRNA identification and analysis

The raw data were first filtered by Trimomatic (v0.36) to remove low-quality reads and adapter sequences, which were then mapped to the *Chlorocebus sabaeus* genome using HISAT2 (v2.2.1). Afterward, transcripts that overlapped with known mRNAs and shorter than 200 bp were discarded. Next, we utilized CNCI (v1.0), CPC (0.9-r2), Pfam-scan (v30), and PLEK (v1.2) to predict the coding ability of transcripts. Transcripts with coding potential predicted by the four tools listed above were discarded, and those without coding potential were classified as lncRNAs. The target genes of lncRNAs were predicted using cis- and trans-regulation analyses [49]. Cufflinks (v2.2.1) was used to calculate the expression level of each transcript by calculating FPKM. DE lncRNAs were selected with fold change > 2 and *P*-value < 0.05 by DESeq R package [48].

### GO and KEGG Enrichment analysis

The DAVID online tool was used to perform Gene Ontology (GO) enrichment analysis and Kyoto Encyclopedia of Genes and Genomes (KEGG) pathway analysis of differentially expressed genes and target genes [50, 51]. GO terms and KEGG pathways with *P*-value < 0.05 were considered to be significantly enriched.

### Construction of lncRNA-miRNA-mRNA network

Based on the expression of different mRNAs, miRNAs, and lncRNAs, Pearson’s correlation coefficient and *P*-value were calculated for miRNA-target pairs. The Pearson correlation coefficient ≥ 0.8 or ≤ -0.8, with *P* < 0.05 was considered statistically significant. MiRanda (v 3.3a) was employed to predict the relationship between miRNAs and mRNAs as well as between lncRNAs and miRNAs. The threshold parameter was set as described previously: S ≥ 150, ΔG ≤  − 30 kcal/mol and strict 5’ seed pairing [52]. Cytoscape (v3.5.1) was used to create and visualize the lncRNA-miRNA-mRNA networks.

### Quantitative real time-PCR (qRT-PCR) verification

To validate the data obtained from RNA-seq, qRT-PCR was performed for the randomly selected immune-related RNAs. Total RNA from PEDV-infected and mock-infected cells was extracted using TRIzol reagent (Invitrogen, USA), and reversely transcribed using the FastKing RT Kit (Tiangen, China). Then, qRT-PCR assays were performed with SYBR Green qPCR Master Mix (Bio-Rad, USA). The β-actin gene and U6 snRNA were selected as the internal control. The relative quantification was determined using the 2^−ΔΔct^ method. The sequence of the primers used in this study is listed in Table S7.

### Statistical analysis

GraphPad Prism 6.0 software was used for statistical analyses. Differences were determined to be statistically significant at values of *P* < 0.05 by Student’s t-test for two groups and one-way ANOVA analysis for more than two groups. (Data are shown as means ± standard deviation (SD). *n* = 3).

## Supplementary Information

**Additional file 1:** **Table S1. **Overview of the RNA sequencing data.

**Additional file 2:** **Table S2. **Alignment information of clean reads with thedatabase.

**Additional file 3:** **Table S3. **Characteristics of the reads from small RNAsequencing libraries.

**Additional file 4:** **Table S4. **Differentially expressed mRNA.

**Additional file 5:** **Table S5. **Differentially expressed miRNA.

**Additional file 6:** **Table S6. **Differentially expressed lncRNA.

**Additional file 7:** **Table S7. **Primers used in this study.

**Additional file 8:** **Figure S1. **Identification of mRNA, miRNA, and lncRNAexpression profiles.

**Additional file 9:** **Figure S2. **Characteristics of DE miRNAs and DE lncRNAsexpression levels.

**Additional file 10:** **Figure S3. **Functional enrichment analysis of DE miRNAs.

**Additional file 11:** **Figure S4. **Functional enrichment analysis of DE lncRNAs.

**Additional file 12:** **Figure S5. **An overview of the top 1000 ceRNA network.

## Data Availability

All raw data has been deposited into the National Center for Biotechnology Information (https://www.ncbi.nlm.nih.gov) under the accession number PRJNA758783.

## Abbreviations

PEDV: Porcine epidemic diarrhea virus
ncRNAs: Non-coding RNAs
miRNA: MicroRNA
lncRNA: Long non-coding RNA
mRNA: Messenger RNA
rRNA: Ribosome RNA
snRNA: Small nuclear RNA
snoRNA: Small nucleolar RNA
tRNA: Transport RNA
ceRNA: Competing endogenous RNA
GO: Gene ontology
KEGG: Kyoto encyclopedia of genes and genomes
DE: Differentially expressed
