# Supplementary material for: Differential expression analysis of mRNAs, lncRNAs, and miRNAs expression profiles and construction of ceRNA networks in PEDV infection
Source: BMC Genomics. 2022 Aug 13;23:586. doi: 10.1186/s12864-022-08805-0 (PMC9375197; doi:10.1186/s12864-022-08805-0)
Supplement: Supplementary file 8 — Additional file 8: Figure S1. Identification of mRNA, miRNA, and lncRNAexpression profiles. [file 12864_2022_8805_MOESM8_ESM.docx]

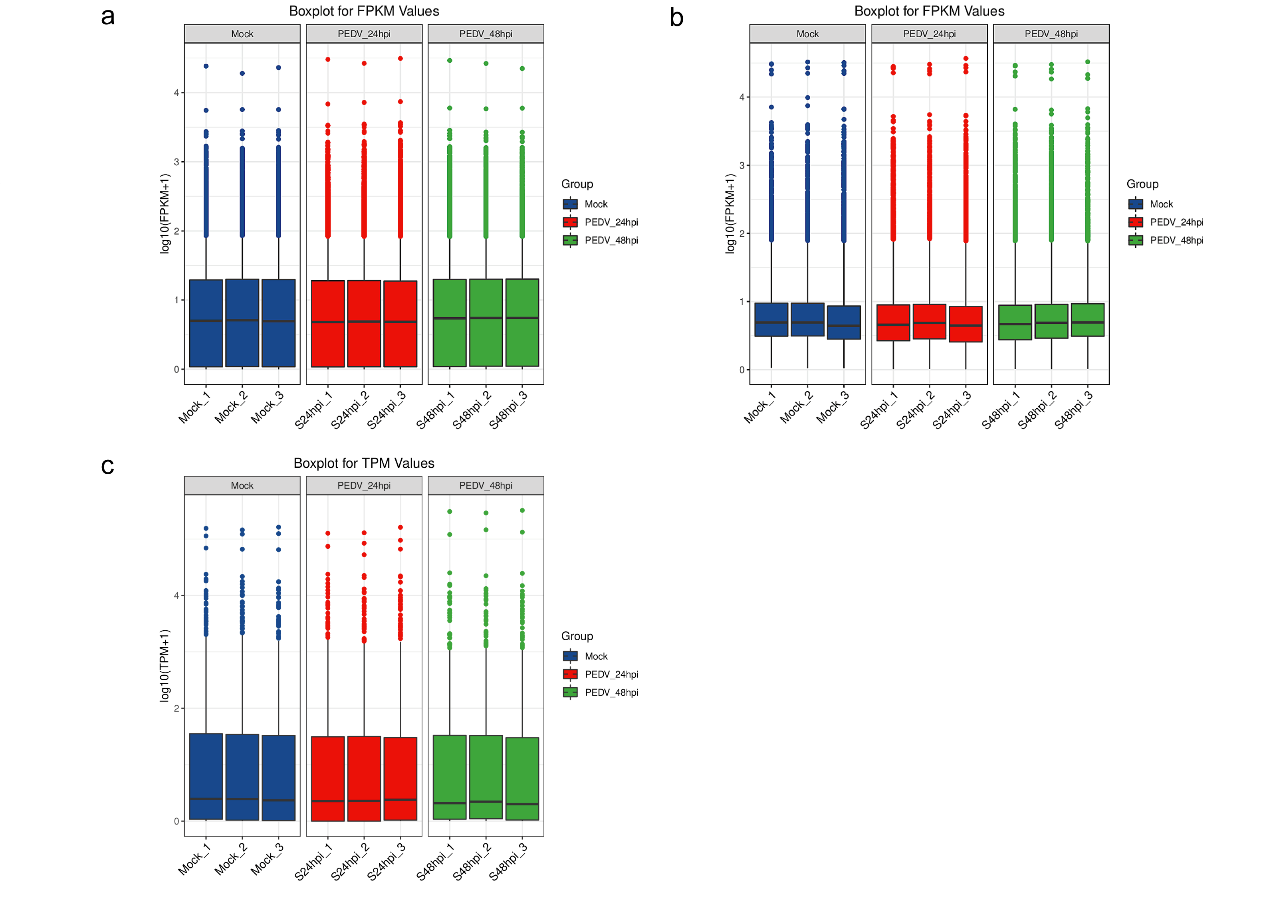


Figure S1 Identification of mRNA, miRNA, and lncRNA expression profiles. Boxplots demonstrating the expression levels (shown in log_10_ (FPKM +1) or log_10_ (TPM +1)) of mRNAs (a), lncRNAs (b) and miRNAs (c) in each sample.
