# Supplementary material for: Differential expression analysis of mRNAs, lncRNAs, and miRNAs expression profiles and construction of ceRNA networks in PEDV infection
Source: BMC Genomics. 2022 Aug 13;23:586. doi: 10.1186/s12864-022-08805-0 (PMC9375197; doi:10.1186/s12864-022-08805-0)
Supplement: Supplementary file 9 — Additional file 9: Figure S2. Characteristics of DE miRNAs and DE lncRNAsexpression levels. [file 12864_2022_8805_MOESM9_ESM.docx]

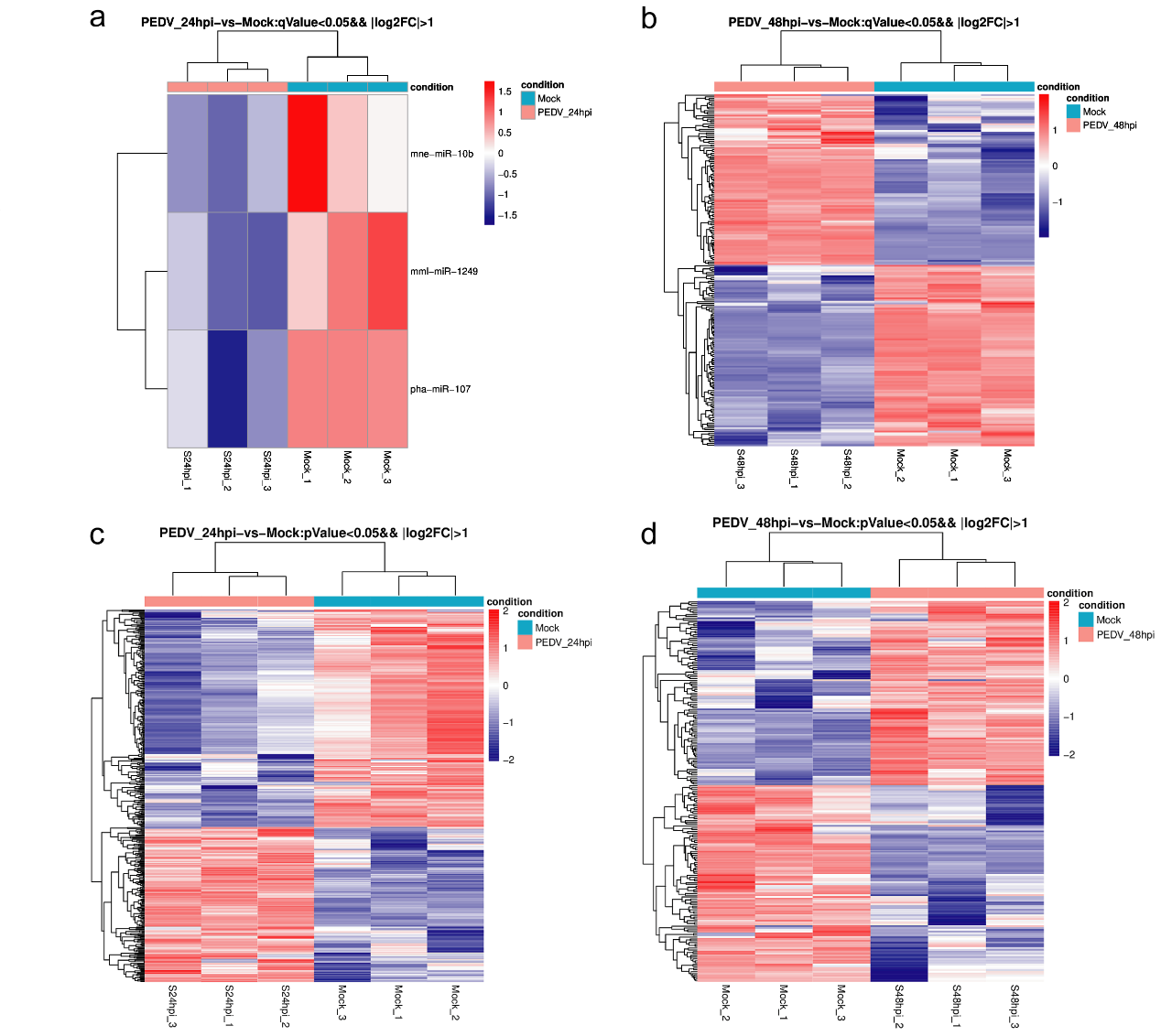


Figure S2 Characteristics of DE miRNAs and DE lncRNAs expression levels. The Hierarchical Cluster Analysis of DE miRNAs (a, b) and DE lncRNAs (c, d). The columns represent individual samples, and rows represent genes with significant expression differences between the two groups.
