# Supplementary material for: Differential expression analysis of mRNAs, lncRNAs, and miRNAs expression profiles and construction of ceRNA networks in PEDV infection
Source: BMC Genomics. 2022 Aug 13;23:586. doi: 10.1186/s12864-022-08805-0 (PMC9375197; doi:10.1186/s12864-022-08805-0)
Supplement: Supplementary file 10 — Additional file 10: Figure S3. Functional enrichment analysis of DE miRNAs. [file 12864_2022_8805_MOESM10_ESM.docx]

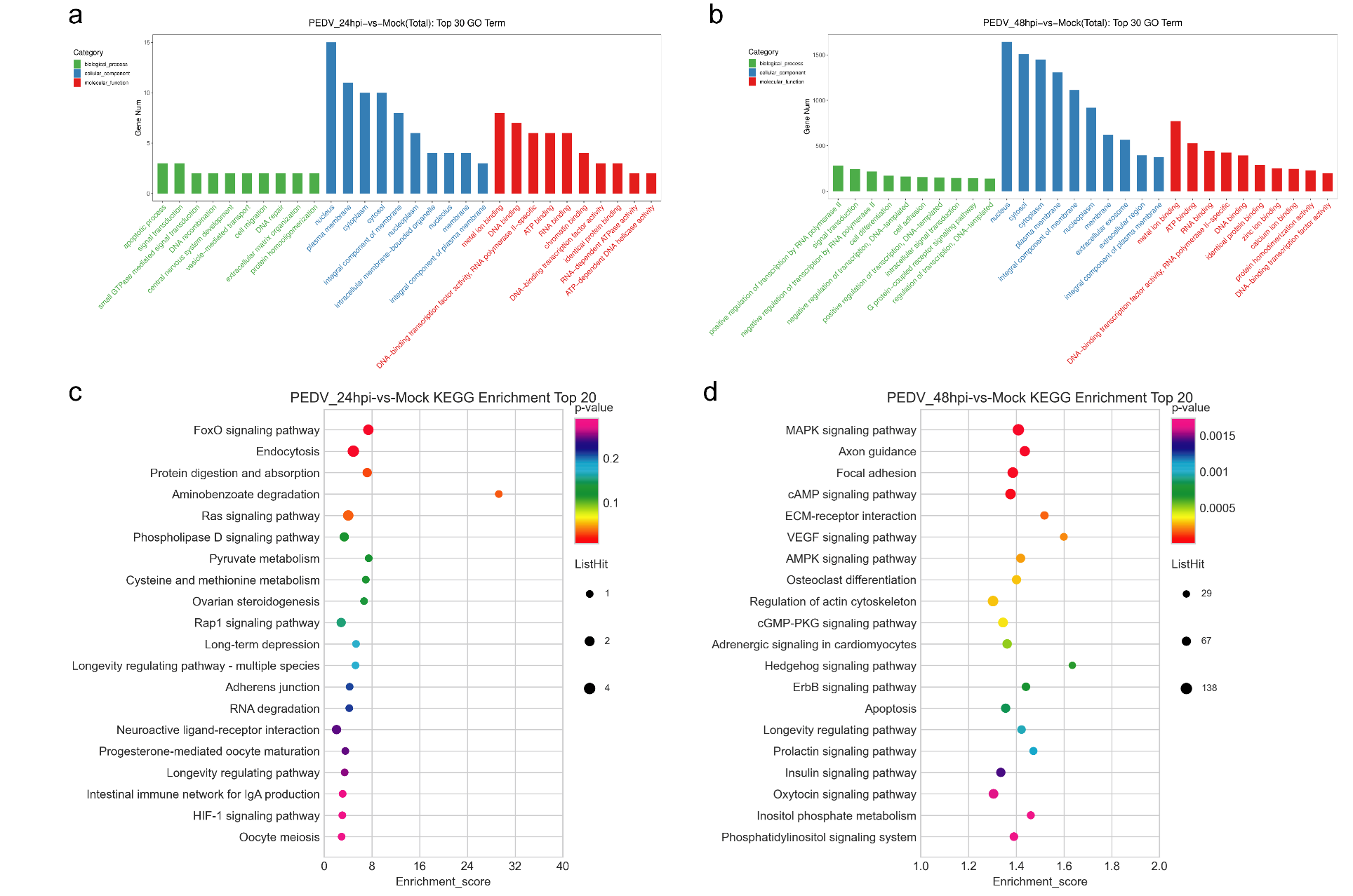


Figure S3 Functional enrichment analysis of DE miRNAs. (a, b) The top 30 significantly enriched GO terms. (c, d) The top 20 significantly enriched KEGG pathways.
