# Supplementary figures and images for: Differential expression analysis of mRNAs, lncRNAs, and miRNAs expression profiles and construction of ceRNA networks in PEDV infection
Source: BMC Genomics. 2022 Aug 13;23:586. doi: 10.1186/s12864-022-08805-0 (PMC9375197; doi:10.1186/s12864-022-08805-0)

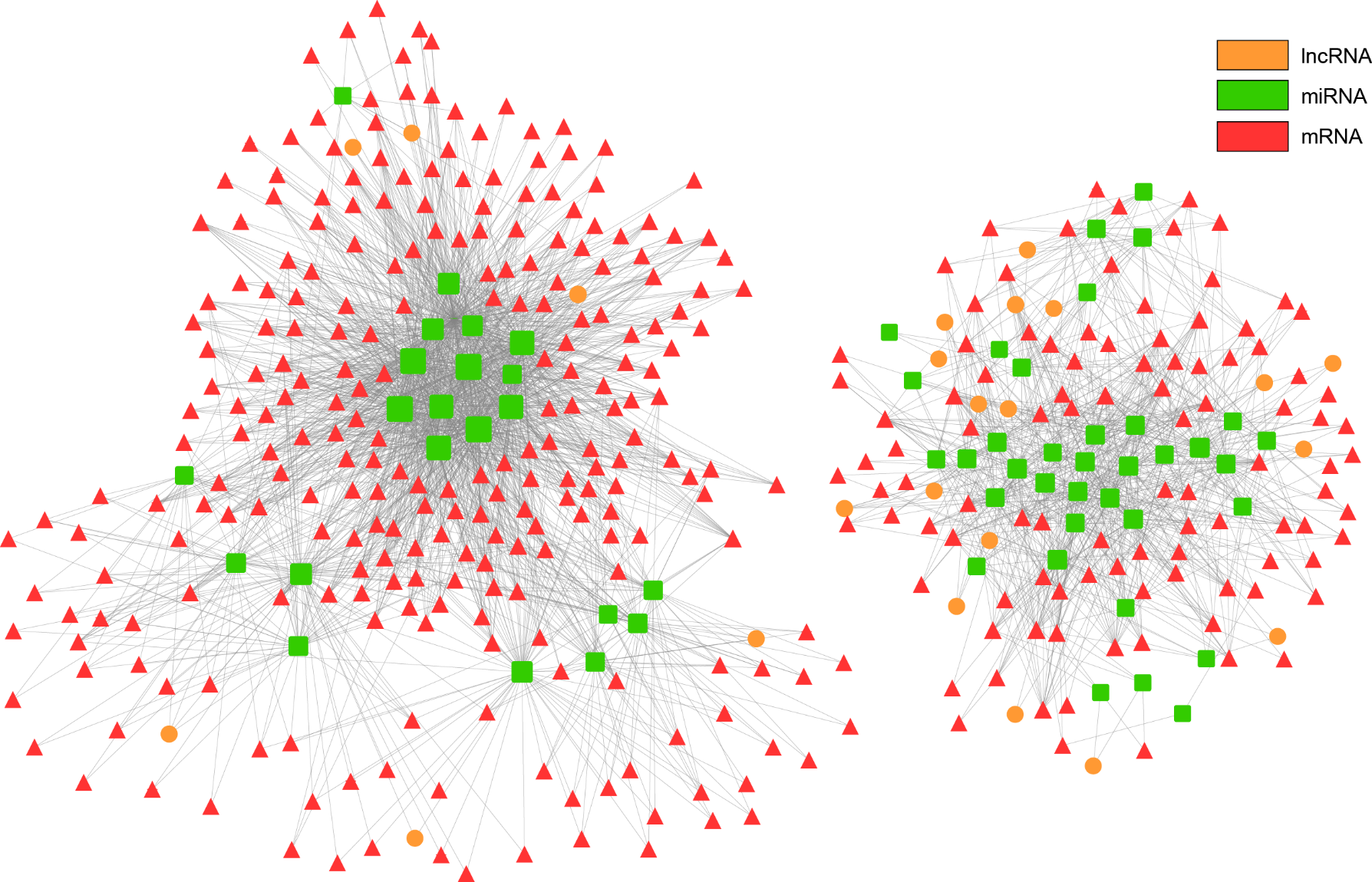


Figure S5 An overview of the top 1000 ceRNA network.

Supplement: Supplementary file 12 — Additional file 12: Figure S5. An overview of the top 1000 ceRNA network. [file 12864_2022_8805_MOESM12_ESM.docx]
